# Kidney transplant tolerance associated with remote autologous mesenchymal stromal cell administration

**DOI:** 10.1002/sctm.19-0185

**Published:** 2019-12-24

**Authors:** Federica Casiraghi, Norberto Perico, Eliana Gotti, Marta Todeschini, Marilena Mister, Monica Cortinovis, Valentina Portalupi, Anna Rita Plati, Flavio Gaspari, Alessandro Villa, Martino Introna, Elena Longhi, Giuseppe Remuzzi

**Affiliations:** ^1^ Istituto di Ricerche Farmacologiche Mario Negri IRCCS Bergamo Italy; ^2^ Unit of Nephrology and Dialysis Azienda Socio‐Sanitaria Territoriale Papa Giovanni XXIII Bergamo Italy; ^3^ G. Lanzani Laboratory of Cell Therapy Azienda Socio‐Sanitaria Territoriale Papa Giovanni XXIII Bergamo Italy; ^4^ Laboratory of Transplant Immunology UOC Coordinamento Trapianti IRCCS Fondazione Ca' Granda – Ospedale Maggiore Policlinico di Milano Milan Italy; ^5^ L. Sacco Department of Biomedical and Clinical Sciences University of Milan Milan Italy

**Keywords:** immunoregulation, immunosuppression withdrawal, kidney transplantation, mesenchymal stromal cells, tolerance

## Abstract

Here we report the case of successful immune tolerance induction in a living‐donor kidney transplant recipient remotely treated with autologous bone marrow‐derived mesenchymal stromal cells (MSC). This case report, which to the best of our knowledge is the first in the world in this setting, provides evidence that the modulation of the host immune system with MSC can enable the safe withdrawal of maintenance immunosuppressive drugs while preserving optimal long‐term kidney allograft function.

## Graphical abstract

This report provides the evidence that autologous BM‐MSC infusion induces a pro‐tolerogenic immune environment, thus allowing complete discontinuation of antirejection drugs in kidney transplantation

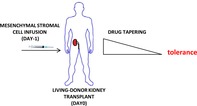

1

**Lessons learned**

- Autologous bone marrow‐derived mesenchymal stromal cells (MSCs) infusion in kidney transplant recipients promoted a sustained and long‐lasting pro‐tolerogenic immune environment. This immune profile was particularly remarkable in a kidney transplant patient.
- This patient was successfully weaned off immunosuppressive drugs and is now 18 months free from antirejection therapy with optimal kidney allograft function.
- This case report provides evidence that MSC could modulate the host immune system, enabling the induction of operational tolerance, and sets the basis for future clinical trials in solid organ transplantation.

**Significance statement**

This case report provides the first evidence that in living‐donor kidney transplantation autologous bone marrow‐derived mesenchymal stromal cells (MSCs) infusion can be associated with safe, complete discontinuation of maintenance antirejection drugs late after transplant, eventually allowing a state of operational tolerance. This case could be also preparatory for future studies to assess whether a panel of noninvasive immunomonitoring tools, in addition to clinical criteria, could identify a pro‐tolerogenic signature after MSC therapy that could eventually help to identify patients who are amenable to safe immunosuppressive drug discontinuation. Further investigations building on this approach are critically needed in living‐donor as well as in deceased donor kidney transplantation.

2

Solid organ transplantation is an established option for many types of end‐stage organ failure. The introduction of new immunosuppressive drugs and biologics has transformed the field of transplantation, leading to significant improvements in the short‐term survival rates of solid organ allografts, including the kidney. Unfortunately, the indispensable long‐term use of immunosuppressive agents results in nonspecific inhibition of the host immune system, as well as off‐target effects, increasing the risk of life‐threatening infections and malignancies,^1^ in addition to cardiovascular and metabolic diseases,^2^ all of which adversely impact allograft function and outcomes. Furthermore, despite their potent effect in inhibiting acute graft rejection, these agents, including the modern, sophisticated, and costly biologics, do not prevent chronic allograft rejection,^3^, ^4^ one of the leading causes of graft failure beyond 1‐year post‐transplantation.

Twenty years after its launch, the Immune Tolerance Network consortium, created to accelerate the clinical development of promising agents for the induction and maintenance of stable, long‐term immune tolerance, falls short of expectations—to successfully and safely achieve tolerance in humans.^5^ Reports of kidney and liver allograft recipients who became spontaneously immunosuppressive‐free have provided the proof of principle that transplantation tolerance can theoretically be achieved.^6^, ^7^ The intentional generation of immune tolerance in kidney transplantation through bone marrow or hematopoietic stem cell/facilitator cell‐based therapies has been reported in a small subset of patients,^8^, ^9^ but there has been a high risk of infections, graft‐vs‐host disease, and malignancies. These significant side effects are inherently associated with the necessary peri‐transplant conditioning regimens adopted in the different cell‐based procedures, making these protocols too risky to justify routine use in patients without malignancies. Although the ongoing development of T regulatory (Treg) cell therapy—as an alternative immunomodulatory cell population that does not require peri‐transplant conditioning regimens—is promising, the ONE study (involving a small number of living‐donor renal transplant recipients) faces major hurdles, such as the potential for bystander global immunosuppression, the lack of effective strategies for expanding antigen‐specific Treg cells ex vivo, and issues related to lineage instability in inflammatory microenvironments.^10^ Interestingly, there are also small clinical trials that are testing the safety and preliminary efficacy of autologous or donor‐derived regulatory dendritic cells in renal and liver transplantation,^11^ but none of the patients have been weaned off the antirejection drugs. Immunomodulatory cell therapy with MSC in organ transplantation, which does not require peri‐transplant conditioning regimens, has reached more advanced stages of clinical development.^11^

Based on our preclinical findings in a cardiac transplant murine model, showing the ability of syngeneic and allogeneic MSC to induce regulatory T‐cell‐mediated tolerance,^12^ in 2009 we first designed and initiated a pilot safety and feasibility clinical study of autologous BM‐derived MSC in living‐donor kidney transplant recipients.^13^ However, the first two patients who received MSC 7 days after transplant experienced transient renal graft dysfunction a few days after MSC infusion.^13^ We ruled out ongoing acute cellular rejection but found histologic evidence of graft inflammation with increased neutrophils and complement C3 deposit (engraftment syndrome).^13^ This observation led us to hypothesize that, in the intragraft subclinical inflammatory environment that occurs in the first few days after transplantation, MSC infused after transplantation may have been recruited primarily to the graft and activated, eventually amplifying the local inflammatory process to the level that affected graft function. We therefore moved back to a murine kidney transplant model and found that a single administration of syngeneic MSC 1 day before but not after renal transplantation avoided the acute deterioration of graft function while maintaining the immunomodulatory tolerogenic effects of the cell therapy.^14^ MSC infused before transplant localized into lymphoid organs, where they promoted early expansion of Treg cells. The clinical protocol was then amended accordingly and MSC infusion was set the day before transplantation. Two additional living‐related donor kidney transplant patients given autologous MSC no longer experienced engraftment syndrome or any other possible cell treatment‐related adverse events thereafter.^15^, ^16^ Other groups have also confirmed the safety and clinical feasibility of autologous or allogeneic MSC‐based therapy in kidney transplantation.^17^, ^18^ However, most of these studies lack mechanistic information on the immunomodulatory properties of MSC treatment and, more importantly, have not yet explored the possibility of inducing long‐term graft tolerance with this cell‐based approach. Here, we present results from the first reported case of a MSC‐treated kidney transplant patient successfully weaned off immunosuppressive drug therapy and describe changes in the host immune microenvironment following cell treatment, a potential mechanism of action through which the modulation of immune regulatory pathways may impact graft rejection and enable the development and retention of a state of donor‐specific graft tolerance.

The patient was a 37‐year‐old male with end‐stage‐renal disease who received a living‐related donor kidney transplant in October 2010 and was enrolled in a pilot safety and feasibility study of pretransplant infusion of autologous ex vivo expanded BM‐derived MSC (http://ClinicalTrials.gov NCT00752479, NCT02012153). He was on peritoneal dialysis as a result of end‐stage renal disease secondary to immunoglobulin (Ig)A nephropathy and received a kidney transplant from his father, mismatched for two human leukocyte antigen (HLA) haplotypes (one on HLA‐A and one HLA‐DR). The cross‐match was negative, as were the antidonor HLA antibodies. The day before transplantation (day −1), the patient was given autologous BM‐MSC (2 × 10^6^ per kg body weight) intravenously. From day 0 to day 6 after transplantation, induction therapy with low‐dose rabbit antithymocyte globulin (RATG) infusion (0.5 mg/kg, i.v. daily) was administered.^19^

Maintenance immunosuppression was with cyclosporine A (CsA, target trough blood levels of 300‐400 ng/mL up to day 7 after surgery, and 100‐150 ng/mL at month 5 after transplantation), mycophenolate mofetil (MMF, 750 mg b.i.d.), and steroids. Five hundred milligrams of methylprednisolone were administered before RATG and continued for 2 more days after transplantation (250 and 125 mg, respectively). Thereafter, oral prednisone (75 mg) was administered, which was progressively tapered and discontinued on day 7 after surgery.^16^ The MSC preparation and release criteria, clinical protocol for MSC treatment, induction and maintenance immunosuppressive therapy, and baseline characteristics are detailed in the Supplemental Online Methods and Figure 1. Following the infusion of BM‐derived MSC, the patient rapidly recovered normal renal function after transplantation and thereafter had an uncomplicated clinical course, with stable graft function for the next 2 years (Figure 1A). At 1 year after transplantation, the surveillance protocol biopsy showed no evidence of subclinical rejection (Banff Score Class 1: Normal biopsy or nonspecific changes, Figure 1D,F). There was no evidence of de novo donor‐specific antibody development. Moreover, during the follow‐up period, he developed a sustained pro‐tolerogenic immune environment, as demonstrated by the high Treg/memory CD8^+^ T‐cell ratio, the expansion of naïve and transitional B cells in the peripheral blood (Figure 2A‐D) and antidonor CD8^+^ T‐cell unresponsiveness on cytotoxicity test (Figure 2E,F).

**Figure 1:**
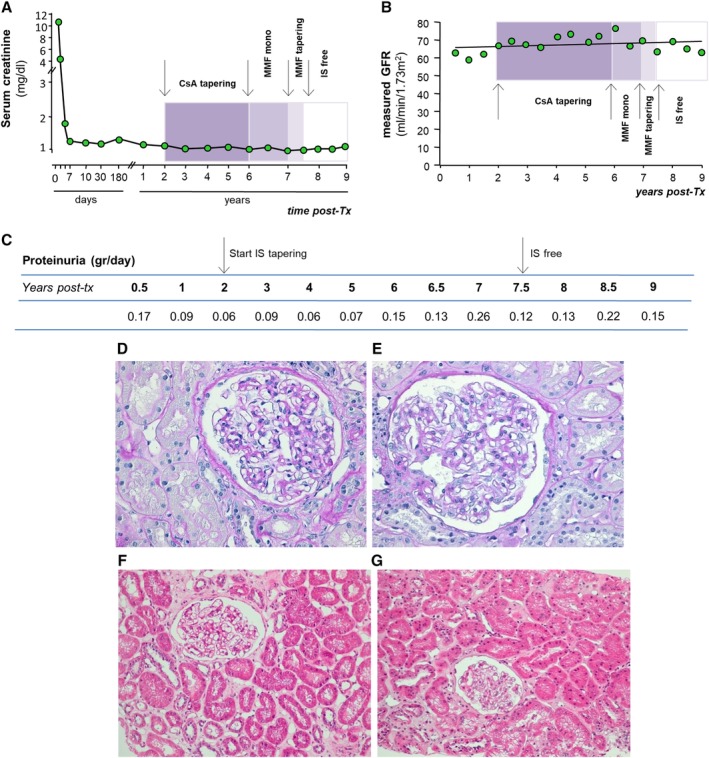
Kidney graft function. A, Post‐transplant course of changes in serum creatinine levels of the patient. Serum creatinine decreased very quickly following kidney transplant and remained constant around the value of 1 mg/dL for the entire 9‐year follow‐up. B, Profile of glomerular filtration rate (GFR), measured by plasma clearance of iohexol every 6 months after transplant, during the long‐term follow‐up. Its slope showed a tendency of GFR to increase over time. Colored boxes highlight the sequential phases of cyclosporine A (CsA) and mycophenolate mofetil (MMF) tapering and discontinuation, indicated by arrows. C, Profile of 24‐hour urinary protein excretion during the post‐transplant period; arrows indicate the start and the end of immunosuppression tapering and discontinuation. D‐G, Light microscopy findings in patient's renal biopsies at 1 year (D,F) and 8 years (E,G) after transplantation. The glomeruli are patent and normocellular and show only a mild increase in mesangial matrix and initial thickening of the Bowman's capsule. The surrounding cortical parenchyma is well preserved and there is no evidence of acute or chronic inflammation, although patchy, mild interstitial fibrosis and moderate atherosclerosis of interlobular arteries can be noted. All these findings are similar in the two protocol biopsies and likely compatible with the age of the kidney donor (65 years). D,E, Periodic acid‐Schiff (PAS), scale bar = 50 μm. F,G, Hematoxylin and eosin, scale bar = 50 μm

**Figure 2:**
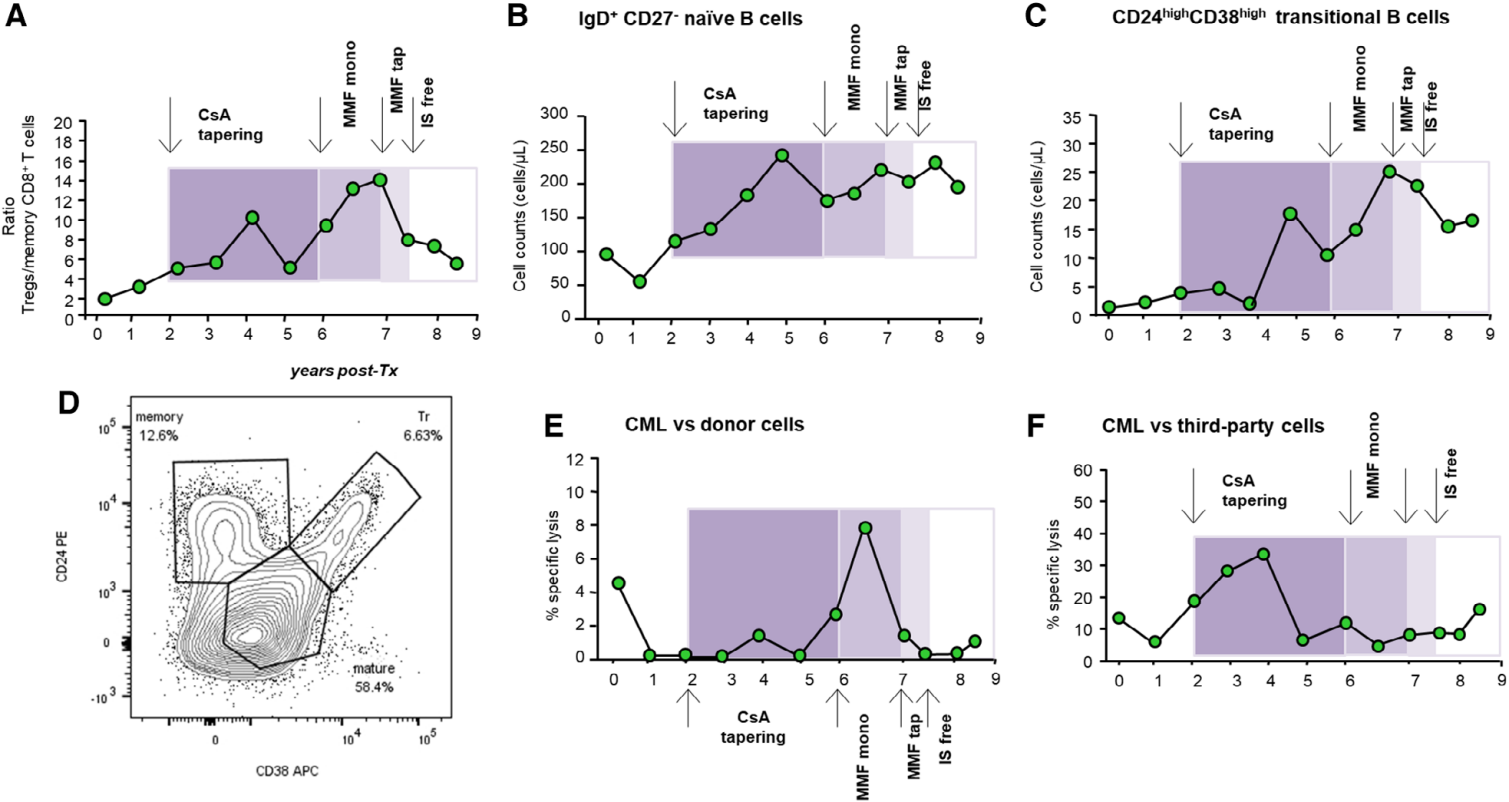
Patient immunological status. Description of the profile of the ratio between percentages of Foxp3^+^CD127^−^CD25^high^CD4^+^ Tregs/CD45RO^+^RA^−^ CD8^+^ memory T cells (see also Supplemental Online Figure 3A,B) (A), of peripheral IgD^+^CD27^−^ naïve (B) and CD24^high^CD38^high^ transitional (C) B‐cell counts during the follow‐up (gating strategy is provided in Supplemental Online Figure 4). Colored boxes highlight the sequential phases of CsA and MMF tapering and discontinuation, indicated by arrows. D, Representative contour plots with outliers of CD38 and CD24 expression on CD19^+^CD3^−^CD45^+^7AAD^−^ B cells from the patient at 8‐year follow‐up. CD38 and CD24 expression divides B cells into memory, mature, and transitional B cells. Percentages of CD8^+^ T‐cell‐mediated lympholysis (CML) of donor (E) or third‐party (F) cells during the different phases of immunosuppressive drug weaning

These clinical and immunological profiles, which revealed a pro‐tolerogenic milieu according to the recent literature,^20^ gave rise to an internal discussion on whether we could pursue our general and long‐term aim of minimizing patient immunosuppressive therapy.^21^ Thus, with the patient's consent, we decided to gradually reduce the dose of immunosuppressive drugs, while closely monitoring renal graft function. Initially, CsA dose was slowly reduced, and it was eventually discontinued 4 years later (Supplemental Online Figure 2A). During this period, graft function remained stable and immunological tests suggested a persistent pro‐tolerogenic milieu. Therefore, after 7 months on MMF monotherapy, this drug was also gradually tapered off and completely withdrawn 10 months later (Supplemental Online Figure 2B). At the time of submission, 18 months had elapsed without immunosuppressive therapy.

Graft function, measured as serum creatinine levels, remained stable during the period when the patient was weaned off immunosuppressives and did not change even during the follow‐up period while free from immunosuppressants (Figure 1A). This pattern was confirmed with more reliable sequential monitoring of graft function, such as glomerular filtration rate (GFR), measured through the plasma clearance of iohexol, which actually demonstrated, on average, a tendency to increase over time (Figure 1B). We cannot rule out, however, the possibility that the rising trend of measured GFR could be attributable, at least in part, to the progressive removal of the intrarenal vasoconstrictive effect of CsA during tapering of this calcineurin inhibitor.^22^ Nevertheless, this approach strengthens our kidney graft function data, because direct measurement of true GFR with this technique avoids bias related to inaccurate GFR estimation using formulas.^23^

Moreover, urinary protein excretion remained within the normal range, both before and after weaning the patient off therapy, clinically ruling out chronic allograft rejection or relapse of his pretransplant glomerulopathy (Figure 1C). Importantly, surveillance graft biopsy specimens were collected at the end of the current monitoring period under free‐immunosuppressive therapy. The histology findings provided evidence of a normal kidney (Banff Score Class 1, Figure 1E,G) with minimal focal tubulo‐interstitial fibrosis, a feature that is substantially similar to that observed in a graft biopsy at 1 year after transplant while the patient was given immunosuppressive drugs (Figure 1D,F) and likely compatible with the advanced age (65 years) of the living kidney donor.

We next assessed whether the favorable clinical course of the kidney allograft was linked to a safeguarded immune tolerogenic milieu promoted by the MSC pretransplant treatment. Analysis of peripheral blood immunophenotype showed that the ratio of Foxp3^+^CD127^−^CD25^high^CD4^+^Treg/CD45RO^+^RA^−^CD8^+^ memory T‐cell percentages was variably elevated during the drug tapering and immunosuppressant‐free periods (Figure 2A). This was the result of the increased percentage of Treg and a concomitant reduction in the percentage of memory CD8^+^ T cells in the blood (Supplemental Online Figure 3A, 3B). Similarly, in the peripheral blood, the number of IgD^+^CD27^−^ naïve B cells (Figure 2B; Supplemental Online Figure 4) and of CD24^high^CD38^high^ transitional B cells (Figure 2C,D; Supplemental Online Figure 4) increased progressively and then stabilized within the same time frame, indicating the presence of a B‐cell profile consistent with that which previously characterized the tolerogenic signature in immunosuppression‐free kidney transplant recipients.^24^ Notably, the patient also exhibited a negligible immune response against the donor (Figure 2E) but not third‐party (Figure 2F) cells in the ex vivo cell‐mediated lympholysis assay, even during the immunosuppressant‐free time period.

What characterizes this tolerant patient who discontinued the immunosuppressive drugs is that he developed a stronger and more sustained pro‐tolerogenic environment than the other three living‐donor transplant patients given autologous MSC before transplant (one) or 7 days after transplant (two) in our center.^15^ Indeed, compared with the other MSC‐treated patients, he exhibited a lower percentage of CD45RO^+^RA^−^ memory CD8^+^ T cells, a higher ratio Treg/memory CD8^+^ T‐cell percentage, and a higher expansion of IgD^+^CD27^−^ naïve B cells and CD24^high^CD38^high^ transitional B cells in the peripheral blood. Because they were participating in a safety and feasibility pilot study, and given the lower pro‐tolerogenic immune milieu, in these other MSC‐treated patients, for safety reasons, we decided not to proceed with tapering/withdrawal of the immunosuppressive drugs. We are also confident that the donor‐specific immune tolerance achieved by our patient is not the result of particular pretransplant characteristics of the recipient, donor, or graft quality, but just the effect of the pro‐tolerogenic environment promoted by MSC infusion. This is supported by previous findings we reported in a control group of kidney transplant patients of comparable mean age at transplantation, similar causes of renal failure, as well as median HLA mismatch with the donor, and donor age, who received induction therapy with low‐dose thymoglobulin alone or in combination with basiliximab, but not MSC.^15^ At variance with the tolerant patient given MSC, the immune profile of Treg/memory CD8^+^ T cells, and naïve and transitional B cells in the peripheral blood, did not change compared with baseline pretransplant values during the same follow‐up period.

As in our tolerant patient, kidney allograft recipients who have been reported to have achieved spontaneous operational tolerance were characterized by an increase in the peripheral blood in the number of naïve and transitional B‐cell subsets.^24^ However, what differentiates and empowers the tolerogenic environment in our patient is the marked reduction in the percentage of CD45RO^+^RA^−^CD8^+^ memory T cells in the peripheral blood, attributed to a specific property that MSC have of inhibiting memory T‐cell expansion/activity,^25^, ^26^, ^27^ which is not shared by the immunosuppressive drugs^28^ taken by the few kidney transplant patients who may then develop spontaneous operational tolerance.

In conclusion, this case provides novel evidence that, in living‐donor kidney transplantation, autologous BM‐derived MSC infusion can be associated with safe, complete discontinuation of maintenance antirejection drugs late after transplant, eventually allowing a state of immune tolerance, and also provide insights into potential mechanisms. Although limited to a single MSC‐treated patient, we are confident that what we observed later after transplantation could bona fide be attributable to the sustained pro‐tolerogenic environment promoted by the single pretransplant cell treatment. Infused MSC, by releasing extracellular vesicles or membrane particles or by undergoing apoptosis, actively engage recipient monocytes/phagocytes and eventually Treg, enabling long‐term immunosuppressive/tolerogenic activity that became self‐sustained after the MSC disappeared.^29^, ^30^

Moreover, this case could be preparatory for future studies aimed at assessing whether a panel of noninvasive immunomonitoring tools, in addition to clinical criteria, could be of help in identifying a pro‐tolerogenic signature after MSC therapy that could eventually help physicians to recognize patients who are amenable to safe immunosuppressive drug discontinuation. Therefore, further investigations building on this approach are critically needed in living‐donor as well as in deceased‐donor kidney transplantation.

## CONFLICT OF INTEREST

The authors indicated no potential conflicts of interest.

## AUTHOR CONTRIBUTIONS

F.C., N.P.: conception and design, data analysis and interpretation, manuscript writing; final approval of manuscript; E.G.: conception and design, data analysis and interpretation, patient monitoring, final approval of manuscript; M.I., G.R.: conception and design, data analysis and interpretation, final approval of manuscript; M.T., M.M., M.C., F.G., E.L.: data analysis and interpretation, collection and/or assembly of data, final approval of manuscript; V.P., A.R.P.: patient monitoring, provision of study samples, final approval of manuscript; A.V.: collection and/or assembly of data, final approval of manuscript.

## Supporting information


**Data S1: Supplementary methods:** Regulatory approvals and patient consent pathway, autologous MSC preparation and expansion, MSC quality controls and release criteria, T‐ and B‐cell phenotypic analysis, cytotoxic T Lymphocyte‐mediated lympholysis, Glomerular Filtration Rate measurement, Ethical Compliance.

**Figure S1** Baseline patient characteristics, protocol for BM collection and autologous MSC expansion, protocol for MSC administration and induction and maintenance immunosuppressive therapy.

**Figure S2** Sequential reduction and the eventual discontinuation of total CsA daily dose and the relative CsA trough levels (C0) from month 27 to month 73 post‐transplant. Scheme of MMF dose reduction from month 80 to month 90, when the patient was immunosuppression free.

**Figure S3** Profile of regulatory T cells (Tregs) and of memeory CD8+ T cells in the patient before, during and after immunosuppressive drug withdrawal and relative representative gating strategies.

**Figure S4** Representative gating strategy for naïve and transitional B cells as contour plots with outliers.

## Data Availability

The data that supports the findings of this study are available in the supplementary material of this article.
