# Supplementary material for: Kidney transplant tolerance associated with remote autologous mesenchymal stromal cell administration
Source: Stem Cells Transl Med. 2019 Dec 24;9(4):427–32. doi: 10.1002/sctm.19-0185 (PMC7103624; doi:10.1002/sctm.19-0185)
Supplement: Supplementary file 1 — Data S1: Supplementary methods: Regulatory approvals and patient consent pathway, autologous MSC preparation and expansion, MSC quality controls and release criteria, T‐ and B‐cell phenotypic analysis, cytotoxic T Lymphocyte‐mediated lympholysis, Glomerular Filtration Rate measurement, Ethical Compliance. [file SCT3-9-427-s001.docx]

**SUPPLEMENTARY METHODS**

**KIDNEY TRANSPLANT TOLERANCE ASSOCIATED WITH REMOTE AUTOLOGOUS MESENCHYMAL STROMAL CELL ADMINISTRATION**

Federica Casiraghi, Norberto Perico, Eliana Gotti, Marta Todeschini, Marilena Mister,

Monica Cortinovis, Valentina Portalupi, Anna Rita Plati, Flavio Gaspari, Alessandro Villa, Martino Introna, Elena Longhi, and Giuseppe Remuzzi

**Regulatory approvals and patient consent pathway**

The patient presented in this article was enrolled in 2010 in a pilot safety and feasibility trial with autologous bone marrow derived mesenchymal stromal cells (MSC) that was ongoing in our center as part of the living-donor kidney transplant program. This trial (Clinical trials.gov NCT00752479, subsequently revised as NCT 02012153) was approved by the Italian Regulatory Authorities (ISS authorization no. 45253(06)-PRE.21-882; and by AIFA on 10 October, 2007 and 30 September, 2013, respectively) and eventually the Local Ethics Committee (authorization no. 352, March 18, 2008 and no. 110/13, November 6, 2013). The patient and the living donor provided written informed consent to participate in this trial.

While enrolled in the trial this patient was expected to participate in the first part of the overall project, namely the safety and biological/mechanistic study, lasting up to 12 months after kidney transplantation. Thereafter, the patient was monitored as part of our routine outpatient transplant clinic, but with special care to monitor any possible changes in his peripheral blood immunophenotype. At 2 years post-transplantation, given the normal graft function and immunological profile – which indicated a pro-tolerogenic environment – we deliberated internally whether we could pursue our general and long-term aim of minimizing patient immunosuppressive therapy [1]. After further discussion with the patient, he consented to starting a program of gradual and sequential reduction of the dose of immunosuppressive drugs, under strict monitoring of graft function. The protocol of CsA and later MMF tapering adopted was, for safety reasons, a more cautions modification than that already approved by Ethics Committee for the second stage of the trial (A pilot efficacy clinical study, authorization no. 352, March 18, 2008 and no. 110/13, November 6, 2013) expected to start when the first feasibility phase is completed.

**Autologous MSC preparation and expansion**

Five months before transplantation the patient underwent cell collection by bone marrow aspiration at the right posterior iliac crest, under local anesthesia.

MSC were processed and expanded *ex-vivo* in good-manufacturing-procedure conditions at the G. Lanzani Centre for Cellular Therapy Azienda Socio Sanitaria Territoriale Papa Giovanni XXIII, Bergamo, Italy (Authorization no. aM-189/2008 AIFA) [2,3].

Nucleated cells were resuspended in alpha-MEM (Gibco-Invitrogen, Carlbad, CA, USA), enriched with 5% human platelet lysate, 50 μg/ml gentamicin (PHT-Pharma, Milan, Italy) and 2UI/ml Heparin (Pharmatex, Milan, Italy) and seeded in one 1-CellSTACK chamber (Corning, NY, USA) at a density of 5x10^5^ TNC/cm^2^. After 2-3 days, the supernatant containing non-adherent cells was removed. 40% medium change was performed twice weekly until approximately 80% confluence was reached. Thirteen days later, cells were detached using animal-origin free TrypLE^TM^ Express (Gibco-Invitrogen). Cells recovered from the first trypsinization were subsequently replated at 300 cells/cm^2^ in 5-CellSTACK chambers (Corning, NY, USA). After a further 8-11 days, cells were harvested and cryopreserved in 90% human AB plasma containing 10% clinical-grade DMSO (WAK-Chemie Medica GmbH, Steinbach, Germany) in clinical-grade cryopreservation bags (Fresenius Medical Care, Bad Homburg, Germany). Aliquots of final cell product and pooled culture supernatants were removed before and after freezing to perform quality control tests (see below). MSCs were suspended in culture medium supplemented with 10% DMSO and stored in liquid nitrogen.

The day before kidney transplantation the MSC were rapidly thawed at 37°C and infused into a peripheral vein of the transplant recipient at the dose of 2x10^6^ cells/kg body weight.

**MSC quality controls and release criteria**

MSC viability, evaluated through Trypan blue dye exclusion, was >80%, and confirmed by 7-amino-actinomycin D (7-AAD) exclusion measured with a FACScan instrument (Becton Dickinson, San José CA, USA). The phenotype of hMSCs was analyzed by staining the cells with anti-CD45-FITC, anti-CD34-PE, anti-CD14-FITC, anti-HLA-DR-FITC, anti-CD90-FITC, anti-CD73-PE, anti-HLA-ABC-PE antibodies (BD Biosciences, San José, CA, USA), anti-CD105-PE (Caltag Laboratories, Burlingame CA) or isotype-matched IgG-FITC and IgG-PE control antibodies (BD Biosciences). Cell products with the following phenotype were considered adequate for release according to guidelines from the International Society for Cellular Therapy: CD45/CD14/CD34 <10%, CD73/CD105/CD90 >70% [4]. Cells were also differentiated into osteogenic and adipogenic lineages, as previously described [2–4]. The cell product was sterile, according to the European Pharmacopeia (Ph.Eur. 2.6.27, Microbiological Control of Cellular Products) using an automated detection system (BactAlert/3D, Biomerieux, Marcy l'Etoile, France) and 14-day incubation. Endotoxin measurement was performed using the Limulus Amebocyte Lysate (PBI S.p.A. Milano, Italy) according to the European Pharmacopeia (Ph.Eur. 2.6.14, Bacterial Endotoxins). Possible Mycoplasma contamination was also detected using the validated culture method and fluorescence method with the Vero indicator fibroblast cell line, and with positive controls, according to the European Pharmacopeia (Ph. Eur. 2.6.7, Mycoplasmas). All these microbiological tests were negative.

Final MSC preparation showed a normal karyotype, evaluated using standard procedures [5].

**T and B cell phenotypic analysis**

Peripheral blood mononuclear cells (PBMC) were isolated through density gradient centrifugation of blood samples. PBMC were stained with fluorochrome-conjugated monoclonal antibodies against human CD3, CD4, CD8, CD45RA, CD45RO, CD25, CD127, FoxP3 for T cell characterization and with mAbs anti-CD19, anti-CD27, anti-CD38, anti-IgD and anti-CD24 for B cell phenotyping. All antibodies were from BD Bioscience (San José, CA, USA). Cells were analyzed with multicolour flow cytometry by FACS Aria and FACS FortessaX20 (BD) and analyzed using FlowJo software.

**Cytotoxic T Lymphocyte-mediated lympholysis**

Cytotoxic T Lymphocyte-mediated lympholysis (CML) was performed as described previously [6]. Briefly, frozen patient PBMC, isolated before and at the different time points post-transplant, were thawed and incubated in mixed lymphocyte reaction with irradiated PBMC (4000 RAD) from the kidney donor or from an unrelated third-party subject in a 24 well-plate (2x10^6^ vs 2x10^6^ cells). At the end of the 5-day incubation, cells were harvested and incubated at a ratio of 50:1 with ^51^Cr-labeled target donor or third-party cells for an additional 4 hours. Results were expressed as % specific lysis.

**Glomerular Filtration Rate measurement**

Following the study protocol, graft function, as glomerular filtration rate (GFR), was measured at 6-month intervals post-transplantation. After a physical examination and under fasting conditions, GFR was measured by injecting 5 ml of iohexol (a non-ionic radiocontrast agent) solution intravenously (Omnipaque 300, GE Healthcare, Milan, Italy) corresponding to 3.235 g of iohexol, as previously described [7]. Thereafter blood samples were collected at 120, 150, 180, 210 and 240 min, since the patient always has values of creatinine clearance >40 ml/min. Plasma levels of iohexol were measured with high-performance liquid chromatography [7].

**Ethical Compliance**

The clinical protocol was approved by the Istituto Superiore di Sanità (ISS, Rome, authorization no. 45253(06)-PRE.21-882 and no. 28689(13)321-1223 and by Agenzia Italiana del Farmaco (AIFA) on 10 October, 2007 and 30 September, 2013, respectively and by the Institutional Review Board of the Ospedali Riuniti/Azienda Socio Sanitaria Territoriale Papa Giovanni XXIII, Bergamo (authorization no. 352, March 18, 2008 and no. 110/13, November 6, 2013). Written informed consent was obtained from the recipient and living donor in accordance with the Declaration of Helsinki.

**References**

1 Sayegh MH, Remuzzi G. Clinical update: immunosuppression minimisation. Lancet 2007;369:1676–1678.

2 Capelli C, Domenghini M, Borleri G, et al. Human platelet lysate allows expansion and clinical grade production of mesenchymal stromal cells from small samples of bone marrow aspirates or marrow filter washouts. Bone Marrow Transplant 2007;40:785–791.

3 Capelli C, Salvade A, Pedrini O, et al. The washouts of discarded bone marrow collection bags and filters are a very abundant source of hMSCs. Cytotherapy 2009;11:403–413.

4 Dominici M, Le Blanc K, Mueller I, et al. Minimal criteria for defining multipotent mesenchymal stromal cells. The International Society for Cellular Therapy position statement. Cytotherapy 2006;8:315–317.

5 Caspersson T, Zech L, Johansson C, et al. Identification of human chromosomes by DNA-binding fluorescent agents. Chromosoma 1970;30:215–227.

6 Rakha A, Todeschini M, Casiraghi F. Assessment of anti-donor T cell proliferation and cytotoxic T lymphocyte-mediated lympholysis in living donor kidney transplant patients. Methods Mol Biol 2014;1213:355–364.

7 Gaspari F, Perico N, Ruggenenti P, et al. Plasma clearance of nonradioactive iohexol as a measure of glomerular filtration rate. J Am Soc Nephrol 1995;6:257–263.
