# Supplementary material for: Kidney transplant tolerance associated with remote autologous mesenchymal stromal cell administration
Source: Stem Cells Transl Med. 2019 Dec 24;9(4):427–32. doi: 10.1002/sctm.19-0185 (PMC7103624; doi:10.1002/sctm.19-0185)
Supplement: Supplementary file 2 — Figure S1 Baseline patient characteristics, protocol for BM collection and autologous MSC expansion, protocol for MSC administration and induction and maintenance immunosuppressive therapy. [file SCT3-9-427-s002.pptx]

## Slide 1
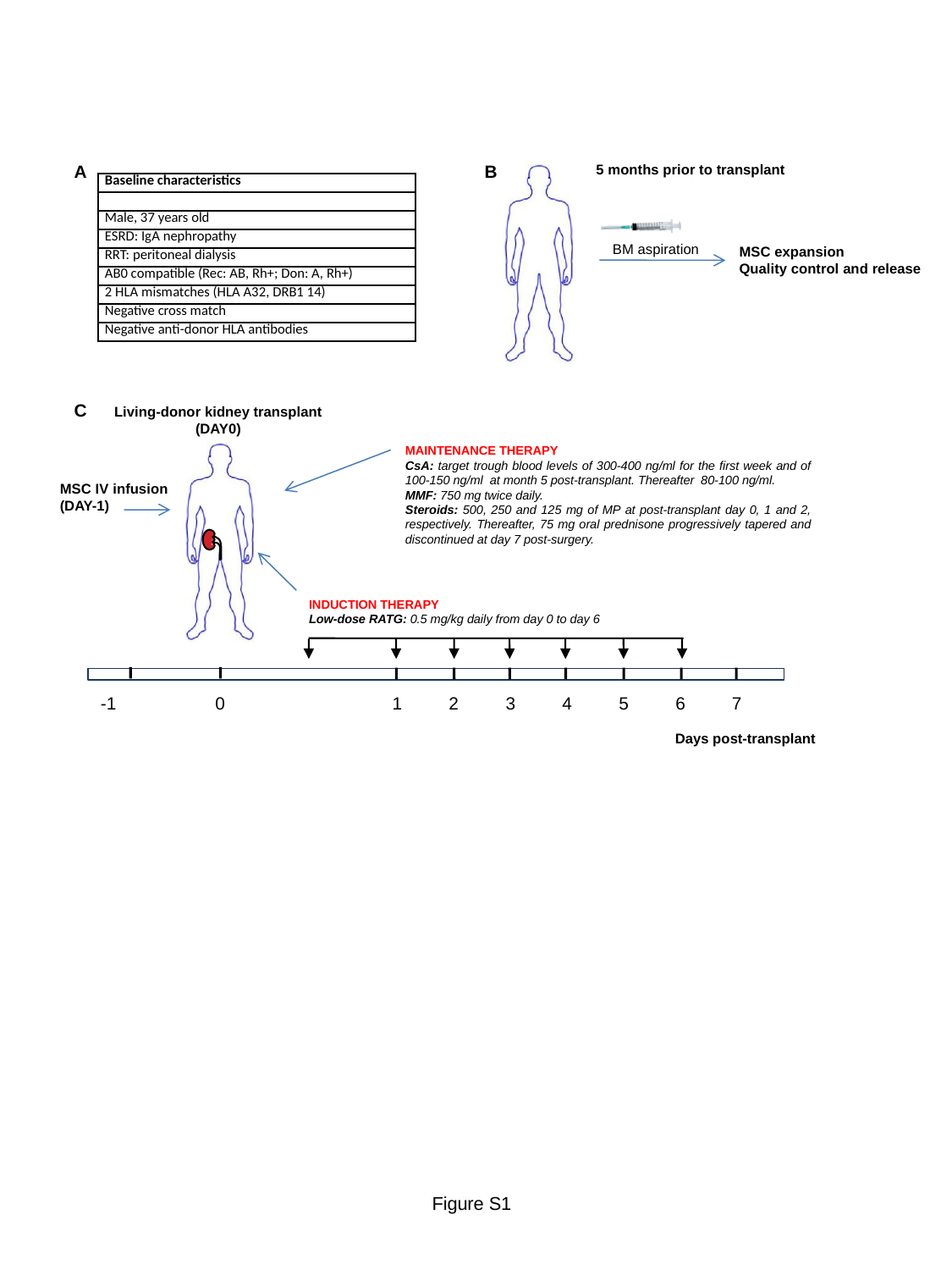

BM aspiration
MSC expansion
Quality control and release
Living-donor kidney transplant
(DAY0)
MAINTENANCE THERAPY
CsA: target trough blood levels of 300-400 ng/ml for the first week and of 100-150 ng/ml at month 5 post-transplant. Thereafter 80-100 ng/ml.
MMF: 750 mg twice daily.
Steroids: 500, 250 and 125 mg of MP at post-transplant day 0, 1 and 2, respectively. Thereafter, 75 mg oral prednisone progressively tapered and discontinued at day 7 post-surgery.
MSC IV infusion
(DAY-1)
INDUCTION THERAPY
Low-dose RATG: 0.5 mg/kg daily from day 0 to day 6
1
2
3
4
5
6
7
-1
0
5 months prior to transplant
A
C
Days post-transplant
B
| Baseline characteristics |
| --- |
| |
| Male, 37 years old |
| ESRD: IgA nephropathy |
| RRT: peritoneal dialysis |
| AB0 compatible (Rec: AB, Rh+; Don: A, Rh+) |
| 2 HLA mismatches (HLA A32, DRB1 14) |
| Negative cross match |
| Negative anti-donor HLA antibodies |
Figure S1
