# Supplementary material for: Kidney transplant tolerance associated with remote autologous mesenchymal stromal cell administration
Source: Stem Cells Transl Med. 2019 Dec 24;9(4):427–32. doi: 10.1002/sctm.19-0185 (PMC7103624; doi:10.1002/sctm.19-0185)
Supplement: Supplementary file 3 — Figure S2 Sequential reduction and the eventual discontinuation of total CsA daily dose and the relative CsA trough levels (C0) from month 27 to month 73 post‐transplant. Scheme of MMF dose reduction from month 80 to month 90, when the patient was immunosuppression free. [file SCT3-9-427-s003.pptx]

## Slide 1
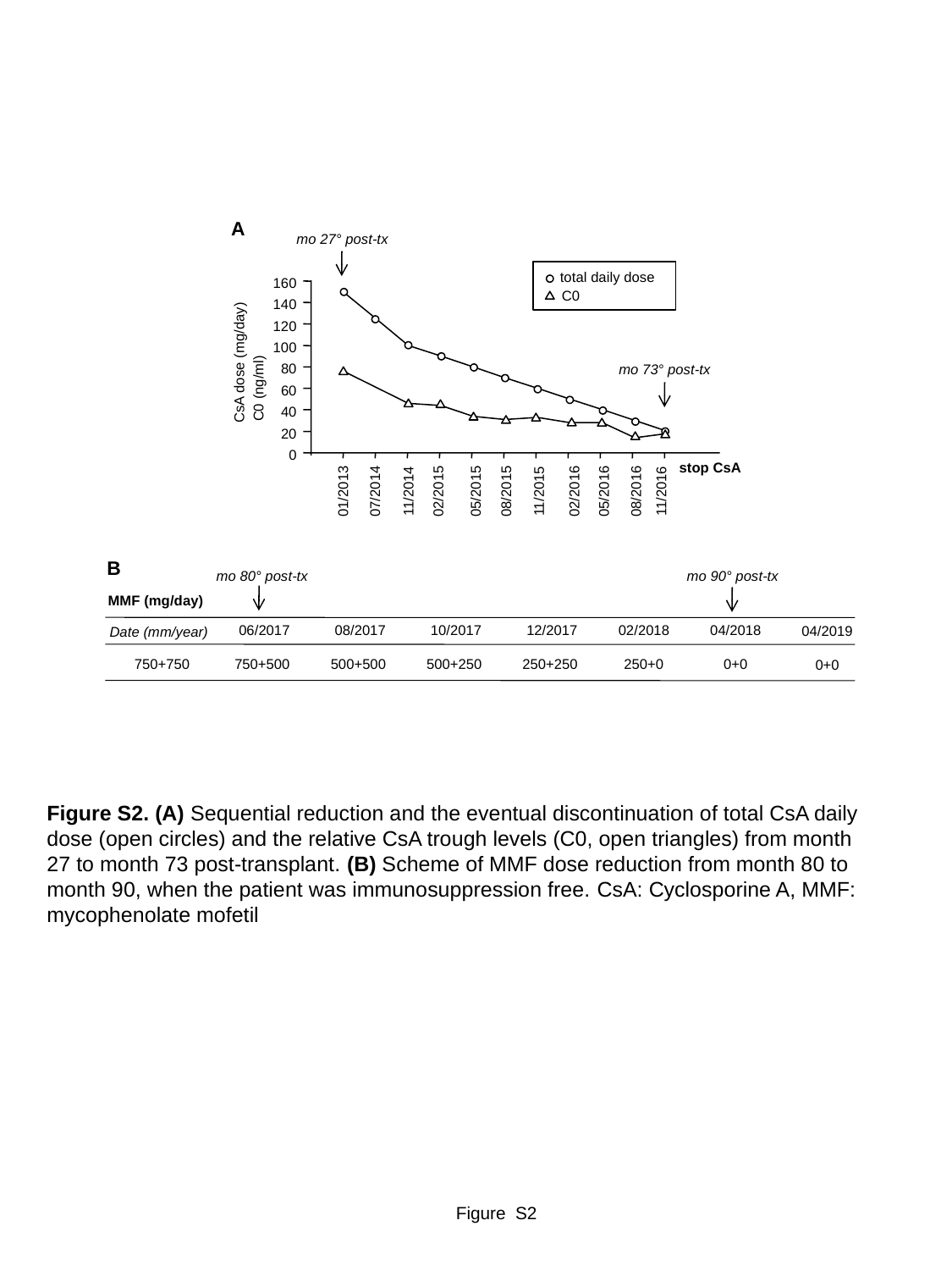

A
mo 27° post-tx
total daily dose
C0
160
140
120
100
80
60
40
20
0
CsA dose (mg/day)
C0 (ng/ml)
mo 73° post-tx
stop CsA
01/2013
07/2014
11/2014
02/2015
05/2015
08/2015
11/2015
02/2016
05/2016
08/2016
11/2016
B
mo 80° post-tx
mo 90° post-tx
MMF (mg/day)
06/2017
750+500
08/2017
500+500
10/2017
500+250
12/2017
250+250
02/2018
250+0
04/2018
0+0
04/2019
0+0
Date (mm/year)
750+750
Figure S2. (A) Sequential reduction and the eventual discontinuation of total CsA daily dose (open circles) and the relative CsA trough levels (C0, open triangles) from month 27 to month 73 post-transplant. (B) Scheme of MMF dose reduction from month 80 to month 90, when the patient was immunosuppression free. CsA: Cyclosporine A, MMF: mycophenolate mofetil
Figure S2
