# Supplementary material for: Kidney transplant tolerance associated with remote autologous mesenchymal stromal cell administration
Source: Stem Cells Transl Med. 2019 Dec 24;9(4):427–32. doi: 10.1002/sctm.19-0185 (PMC7103624; doi:10.1002/sctm.19-0185)
Supplement: Supplementary file 4 — Figure S3 Profile of regulatory T cells (Tregs) and of memeory CD8+ T cells in the patient before, during and after immunosuppressive drug withdrawal and relative representative gating strategies. [file SCT3-9-427-s004.pptx]

## Slide 1
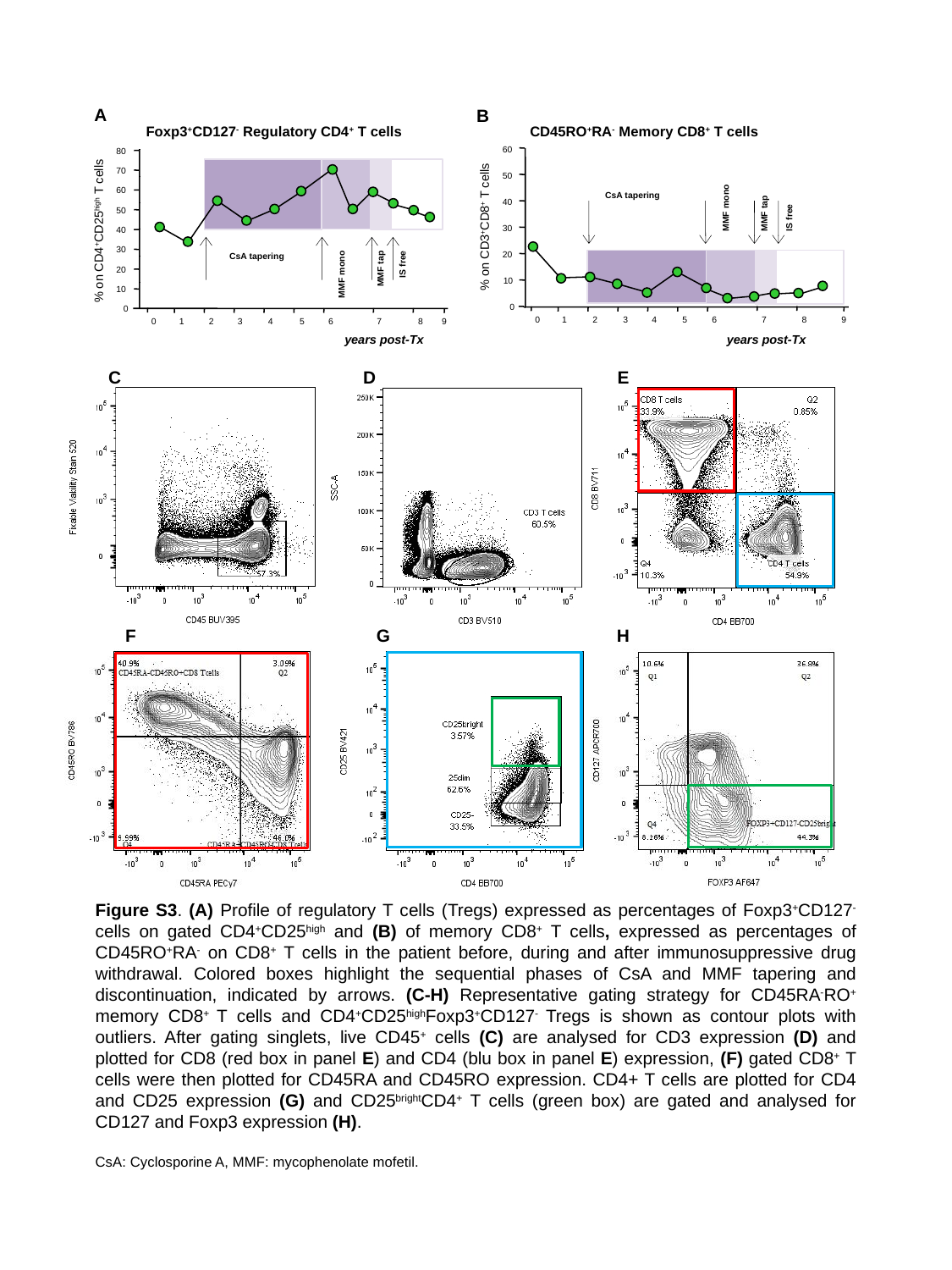

A
Foxp3+CD127- Regulatory CD4+ T cells
80
70
60
50
% on CD4+CD25high T cells
40
30
CsA tapering
20
IS free
MMF tap
MMF mono
10
0
0
1
3
4
5
6
7
8
9
2
years post-Tx
B
CD45RO+RA- Memory CD8+ T cells
60
50
40
30
20
10
0
MMF tap
MMF mono
IS free
CsA tapering
% on CD3+CD8+ T cells
9
0
1
2
3
4
5
6
7
8
years post-Tx
C
D
E
F
G
H
Figure S3. (A) Profile of regulatory T cells (Tregs) expressed as percentages of Foxp3+CD127- cells on gated CD4+CD25high and (B) of memory CD8+ T cells, expressed as percentages of CD45RO+RA- on CD8+ T cells in the patient before, during and after immunosuppressive drug withdrawal. Colored boxes highlight the sequential phases of CsA and MMF tapering and discontinuation, indicated by arrows. (C-H) Representative gating strategy for CD45RA-RO+ memory CD8+ T cells and CD4+CD25highFoxp3+CD127- Tregs is shown as contour plots with outliers. After gating singlets, live CD45+ cells (C) are analysed for CD3 expression (D) and plotted for CD8 (red box in panel E) and CD4 (blu box in panel E) expression, (F) gated CD8+ T cells were then plotted for CD45RA and CD45RO expression. CD4+ T cells are plotted for CD4 and CD25 expression (G) and CD25brightCD4+ T cells (green box) are gated and analysed for CD127 and Foxp3 expression (H).
CsA: Cyclosporine A, MMF: mycophenolate mofetil.
