# Supplementary material for: Kidney transplant tolerance associated with remote autologous mesenchymal stromal cell administration
Source: Stem Cells Transl Med. 2019 Dec 24;9(4):427–32. doi: 10.1002/sctm.19-0185 (PMC7103624; doi:10.1002/sctm.19-0185)
Supplement: Supplementary file 5 — Figure S4 Representative gating strategy for naïve and transitional B cells as contour plots with outliers. [file SCT3-9-427-s005.pptx]

## Slide 1
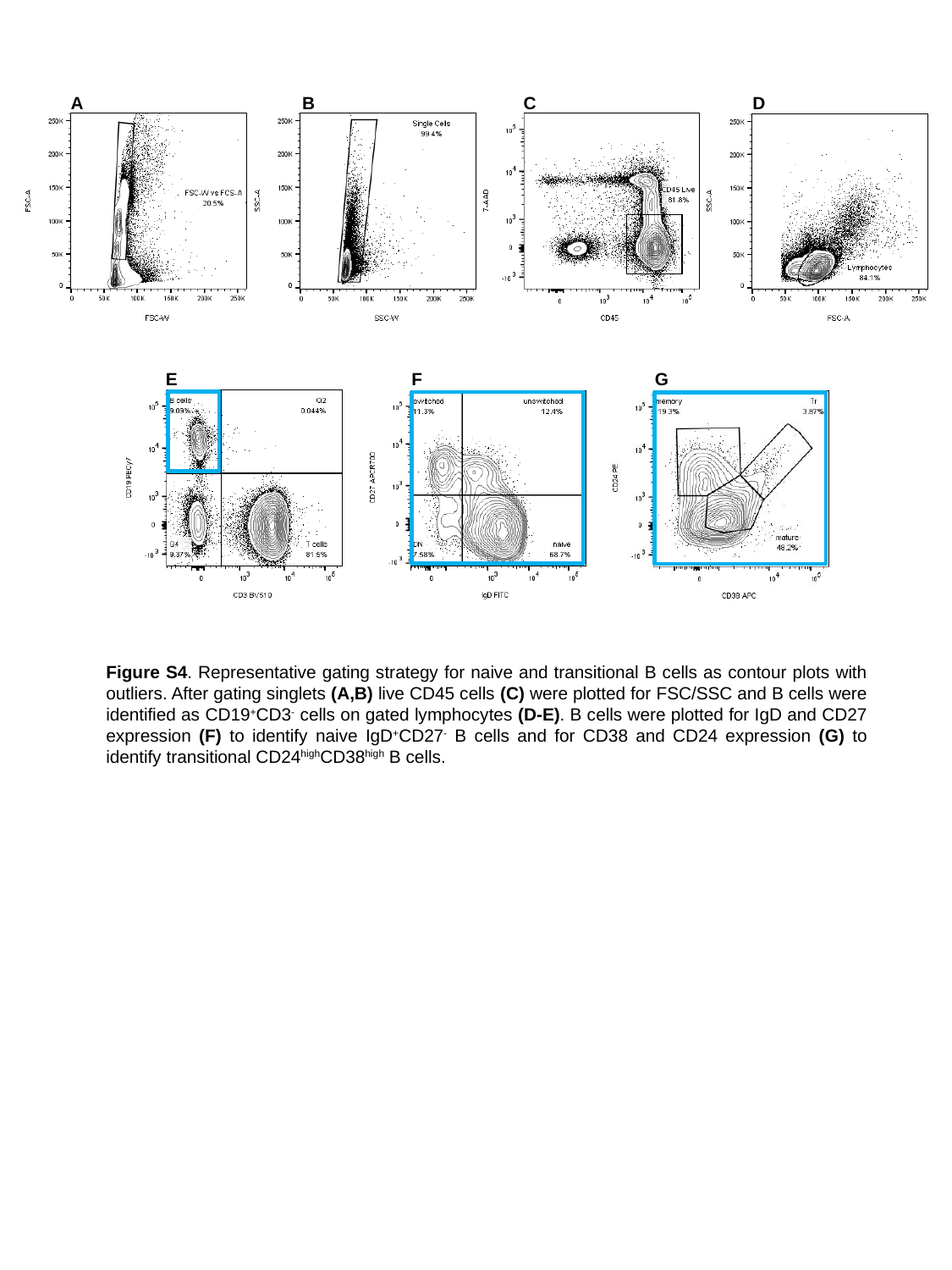

A
B
C
D
E
F
G
Figure S4. Representative gating strategy for naive and transitional B cells as contour plots with outliers. After gating singlets (A,B) live CD45 cells (C) were plotted for FSC/SSC and B cells were identified as CD19+CD3- cells on gated lymphocytes (D-E). B cells were plotted for IgD and CD27 expression (F) to identify naive IgD+CD27- B cells and for CD38 and CD24 expression (G) to identify transitional CD24highCD38high B cells.
